# Prognostic factors identification using machine learning in temporomandibular disorder treatment responders

**DOI:** 10.22514/jofph.2025.076

**Published:** 2025-12-12

**Authors:** Chollada Chamnanmanoontham, Thanaphon Tangchoopong, Jarin Paphangkorakit, Supanigar Ruangsri, Teekayu P. Jorns

**Affiliations:** ^1^Department of Oral Biomedical Sciences, Faculty of Dentistry, Khon Kaen University, 40002 Khon Kaen, Thailand; ^2^College of Computing, Khon Kaen University, 40002 Khon Kaen, Thailand

**Keywords:** Machine learning, Model selection, Prognostic factors, Temporomandibular disorders, Treatment outcome

## Abstract

**Background**: Temporomandibular disorders (TMD) are complex chronic conditions that significantly impair quality of life and impose a considerable social and economic burden. Although various treatment strategies have been developed, the prognostic factors influencing therapeutic outcomes remain poorly defined. This study aimed to identify relevant prognostic factors for treatment response using machine learning methods, with patient readiness for discharge serving as the primary outcome. **Methods**: A total of 1050 medical records from patients diagnosed with TMD and treated at the Orofacial Pain and Dental Sleep Medicine clinic, between January 2018 and June 2023, were retrospectively analyzed with a follow-up period of one year. Twenty-six clinical and demographic variables were initially extracted and preprocessed using one-hot encoding. After the removal of highly correlated variables, the final dataset comprised 36 features derived from encoded categorical variables. Seven machine learning algorithms, namely Decision Tree, Random Forest, Logistic Regression, K-Nearest Neighbors (KNN), Support Vector Classifier (SVC), Gradient Boosting, and Extreme Gradient Boosting (XGBoost), were trained to predict discharge readiness. Model performance was evaluated using accuracy, precision, recall, F1 score, and the receiver operating characteristic-area under the curve (ROC-AUC). Feature importance was assessed using information gain and Shapley Additive exPlanations (SHAP) to interpret model predictions. **Results**: The Random Forest model demonstrated superior performance in predicting readiness for discharge, with an accuracy of 0.7901, precision of 0.8611, recall of 0.8943, F1 score of 0.7901, and ROC-AUC of 0.6442. SHAP analysis identified onset duration, pain severity, anxiety level, presence of neck pain, and body mass index (BMI) as the most influential predictors. **Conclusions**: The identified prognostic factors highlight the multidimensional nature of TMD and support their relevance in guiding patient-specific management strategies. The integration of machine learning approaches may enhance clinical decision-making and contribute to the development of more personalized and effective treatment pathways for TMD.

## 1. Introduction

Temporomandibular disorders (TMD) represent a complex clinical syndrome characterized by persistent dysfunction of the masticatory musculature, the temporomandibular joint (TMJ), and related craniofacial structures [1]. These disorders are typically manifested by chronic orofacial pain, limited mandibular mobility, and joint sounds, all of which collectively impair functional capacity and reduce quality of life. A defining clinical feature of TMD is its chronic and fluctuating nature, which poses substantial challenges for both clinical management and the efficient allocation of healthcare resources [2]. It also has been reported that approximately one-third of affected individuals experience persistent symptoms for an average duration of two to three years at follow-up [3]. In response to these complexities, current paradigms in TMD pain management have increasingly emphasized personalized patient-centered therapeutic strategies to promote patient self-management and achieve sustainable functional recovery over the long term [4].

Achieving a favorable response to treatment remains the primary objective in the management of TMD. Treatment responders are defined as individuals who exhibit clinically meaningful improvement following a specific intervention [5]. The evaluation of treatment response depends on the therapeutic goals and intended outcomes, and typically involves multiple criteria. Commonly used measures include reductions in pain intensity, restoration of jaw mobility, and improvements in psychological and emotional well-being [5]. In addition to these parameters, readiness for discharge, characterized by the patient’s ability to resume normal physical activities or occupational roles, has been proposed as a relevant outcome measure [4, 6]. When assessed in conjunction with clinical findings and patient-reported outcomes, discharge readiness offers a comprehensive perspective on the overall treatment efficacy. Although literature has examined prognostic indicators, such as low back pain, in chronic musculoskeletal pain conditions, there remains a substantial gap in establishing standardized discharge criteria for TMD [4]. Current clinical perspectives emphasize that decisions regarding patient discharge should be guided by a multifactorial assessment, incorporating parameters such as overall health status, therapeutic expectations, and individual self-efficacy [4]. Moreover, both TMD management guidelines and national survey data recommend a structured follow-up period of up to one year, which is considered essential for adequately evaluating patient coping mechanisms and the sustainability of treatment outcomes [4, 7].

Despite notable technological advancements, including the increasing integration of artificial intelligence into diagnostic and classification processes [1, 8] as well as the identification of risk factors for TMD onset and persistence [9], effective management of TMD remains a significant clinical challenge. This ongoing epidemiological burden underscores the need for robust secondary prevention strategies. Machine learning has emerged as a promising approach for predicting discharge readiness in patients with chronic musculoskeletal pain, offering the capacity to analyze complex health data and behavioral patterns [10]. To better elucidate the complexity of TMD and identify the most informative predictors, two feature importance techniques were employed. The first, information gain analysis, is a commonly used method that offers computational efficiency and simplicity in determining the relative contribution of each variable [11]. The second, Shapley Additive exPlanations (SHAP) values, provides a more sophisticated and interpretable framework by quantifying the impact of each feature on individual model predictions. Unlike traditional ranking methods, SHAP assigns importance scores that reflect the marginal contribution of each feature across all possible model outcomes, thereby offering a more comprehensive understanding of feature relevance [12].

The objectives of the present study were to: (1) identify key prognostic factors associated with treatment response, as defined by discharge status at a one-year follow-up, using only baseline clinical and demographic data collected during the patients’ initial visits; and (2) evaluate the predictive performance of various machine learning algorithms in predicting readiness for discharge. The application of machine learning to a large clinical dataset from a specialized orofacial pain center may elucidate the underlying complexity of TMD patient profiles. Ultimately, these insights may inform the development of personalized treatment strategies and contribute to more efficient healthcare resource utilization.

## 2. Materials and methods

### 2.1 Study design and population

This retrospective cohort study was conducted through a review of electronic medical records retrieved from the Hospital Information System for patients diagnosed with TMD and treated at the Orofacial Pain and Dental Sleep Medicine clinic, Faculty of Dentistry, Khon Kaen University, between 01 January 2018 and 30 June 2023. The inclusion criteria were: (1) patients diagnosed with TMD based on the Diagnostic Criteria for Temporomandibular Disorders (DC/TMD) [13] by qualified dental professionals, using the International Classification of Diseases, Tenth Revision (ICD-10) [14]; (2) patients who initiated treatment during the defined study period; and (3) patients with available treatment records for a minimum of one year of follow-up, with final documentation completed no later than 30 June 2024. The exclusion criteria were: (1) patients presenting with concurrent neuropathic or idiopathic orofacial pain conditions such as trigeminal neuralgia or burning mouth syndrome; (2) individuals with TMD secondary to movement disorders, muscular hypertrophy, fractures, or soft tissue lesions; and (3) cases with incomplete demographic or clinical data or patients who were lost to follow-up or could not establish further contact.

### 2.2 Data collection

A total of 1237 patient records were initially retrieved based on the following ICD-10 diagnostic codes: M26.62 (TMJ pain), K07.60 (TMD), K07.61 (TMJ disc-condyle complex disorders), K07.62 (TMJ subluxation), M19.9 (degenerative joint disease), M79.0 (myofascial pain), R25.2 (muscle cramp and spasm), and M27.8 (coronoid hyperplasia). After applying the predefined inclusion and exclusion criteria, a final cohort comprising 1050 eligible patients was established. Clinical information was systematically extracted and manually text-mined from the electronic medical records corresponding to the baseline visit only. All data were subsequently de-identified using a standardized case record form created in Microsoft Excel 365 (Version 2406, Microsoft Corp., Redmond, WA, USA). Initially, 26 demographic and clinical variables were collected. Categorical variables were processed using one-hot encoding, which expanded the number of variables to 36. Variables demonstrating high intercorrelation (correlation coefficient >0.7) were removed to reduce multicollinearity and improve the robustness of subsequent predictive analyses. All demographic, clinical, diagnostic, and outcome variables that were collected and analyzed are summarized in Table 1 (Ref. [4, 15, 16, 17, 18, 19, 20, 21, 22, 23, 24]).

**Table 1.** Summary of collected variables and outcome measurements for each patient.

Variable		Outcome measures	Description
Demographic data			
	Gender	1 = male; 2 = female	Biological sex
	Age	Integer number	Patient’s full year of birth
	Occupation	0 = student/no job; 1 = managers; 2 = professionals; 3 = technicians; 4 = clerical workers; 5 = service workers; 6 = agricultural workers; 7 = craft workers; 8 = machine operators; 9 = elementary workers; 10 = armed forces	Classified according to the International Standard Classification of Occupations-08 (ISCO-08) [15]
Clinical data			
	Numeric rating scale	Score range 0–10: 0 = no pain; 10 = worst possible pain, NRS ≥4 used as the cutoff for significant pain [16]	Numeric rating scale relating to worst pain in the past
	Anxiety	Score range 0–21: 0 = no symptoms; 21 = presence of all assessed anxiety symptom, 0–7 no signs of anxiety [17]	Anxiety score, extracting from Thai HADS [17]
	Depression	Score range 0–21: 0 = no symptoms; 21 = presence of all assessed depression symptom, scores 0–7 no signs of depression [17]	Depression score, extracting from Thai HADS [17]
	Stress	Score range 0–60: 0 = no symptoms; 60 = presence of all assessed stress symptom, score 0–17 = within normal range	Stress score from Thai stress test questionnaire (ST-5) [18]
	Body mass index	Numerical value with two decimal places, 18.5 ≤ BMI < 25 indicates normal weight [19]	Calculated as weight in kilograms divided by height in meters squared
	Sleep problem	0 = no; 1 = yes	Includes prolonged sleep latency (>30 minutes), difficulty in maintaining sleep, or poor sleep quality
	Psychiatric problem	0 = no; 1 = yes	Current psychiatric treatment
	Clenching	0 = no; 1 = yes	Biting on one’s own teeth or freezing the jaw while awake [20]
	Bruxism	0 = no; 1 = yes	Self-reported or observed teeth grinding during sleep [20]
	Onset duration	Numerical value with t decimal places	Duration from symptom onset to clinical presentation, measured in months
	Neck pain	0 = no; 1 = yes	Neck pain experienced in the past 30 days
	Back pain	0 = no; 1 = yes	Lower back pain experienced in the past 30 days
	Poor posture	0 = no; 1 = yes	Habitual or observed abnormal posture
	Irritable bowel syndrome	0 = no; 1 = yes	Presence of abdominal pain, bloating, diarrhea or constipation [21]
	Itchy skin	0 = no; 1 = yes	Irritated sensation prompting scratch of the affected area
	Tinnitus	0 = no; 1 = yes	Ringing or other noise in the ears
	Tension-type headache	0 = no; 1 = yes	Bilateral pressure-type pain in the temples or occipital areas [22]
	Migraine	0 = no; 1 = yes	Unilateral, throbbing headache with or without aura [22]
	Trauma event	0 = no; 1 = yes	History of physical trauma to the head or neck [23]
	Hypertension	0 = no; 1 = yes	History of physician-diagnosed hypertension
	Thyroid disease	0 = no; 1 = yes	History of thyroid dysfunction [24]
	Exercise	0 = no; 1 = yes	Regular general physical exercise performed as part of the patient’s daily routine
	Splint use	0 = no; 1 = yes	Day and/or night occlusal splint use before study enrollment
Diagnosis			
	Diagnosis	1 = pain-related conditions; 2 = structural abnormalities; 3 = combined conditions	Pain-related conditions: DC/TMD diagnoses primarily associated with pain symptoms
			Structural abnormalities: DC/TMD diagnoses involving masticatory structural changes without any pain
			Combined group: DC/TMD diagnoses presenting both pain-related conditions and structural abnormalities
Outcomes			
	Discharge	0 = undischarged; 1 = discharged	Undischarged: extended temporomandibular disorders treatment beyond one year
			Discharged: achieved self-management capability within one year [4]

*DC/TMD: Diagnostic Criteria for Temporomandibular Disorders; Thai HADS: Thai version of the Hospital Anxiety and Depression Scale; NRS: Numeric Rating Scale.*

#### 2.2.1 Demographic variables

Gender was recorded as a binary variable based on biological sex. Age was documented as an integer indicating the patient’s complete years at the time of the initial visit, and patients of all age groups were included in the study. Occupation was classified according to the International Standard Classification of Occupations-08 (ISCO-08) [15].

#### 2.2.2 Clinical parameters

Quantitative clinical parameters included the assessment of pain intensity using an 11-point numeric rating scale (NRS), where 0 represented the absence of pain and 10 indicated the worst imaginable pain. An NRS score of ≥4, denoting moderate to severe pain, was used as the threshold for determining the need for additional anesthesia [16]. Psychological status was evaluated using the validated Thai version of the Hospital Anxiety and Depression Scale (Thai HADS), which comprises two subscales (anxiety and depression), each ranging from 0 to 21. Scores between 0 and 7 on each subscale were considered to indicate no clinically significant signs of anxiety or depression [17]. Stress levels were assessed using the Thai stress test questionnaire (ST-5), with total scores ranging from 0 to 60, where scores from 0 to 17 were classified as within the normal range [18]. Body mass index (BMI) was calculated, and the duration of pain onset, in months, was recorded.

Additionally, patients provided self-reported information on various clinical parameters, such as sleep problems including abnormal sleep latency (more than 30 minutes), inability to maintain sleep throughout the night or lack of sleep quality (individual’s self-dissatisfaction with all aspects of the sleep experience) [25], current or history of psychiatric treatment, the presence of awake clenching, sleep bruxism, current occlusal splint use, neck pain, lower back pain, poor posture, irritable bowel symptoms, itchy skin, tinnitus, tension-type headache, migraines, history of head and neck trauma, hypertension, thyroid disease, and routine general physical exercise. Patients’ responses were recorded as binary variables (yes or no) to indicate the presence or absence of these factors.

#### 2.2.3 Diagnostic categorization

The diagnostic categorization was structured into three main groups: pain-related conditions, structural abnormalities, and a combined category. Diagnoses extracted from the medical records were reclassified according to the DC/TMD criteria. The pain-related group included TMJ pain, masticatory muscle pain, contracture, and headaches attributed to TMD. Structural abnormalities encompassed disc-condyle complex disorders, osteoarthrosis, and coronoid hyperplasia in the absence of pain. The combined group included cases presenting with both pain-related symptoms and structural abnormalities, such as joint disorders with concurrent pain, osteoarthritis, and coronoid hyperplasia associated with pain.

#### 2.2.4 Treatment outcome

Treatment outcomes were evaluated based on patient discharge status and were categorized into two groups: discharged and undischarged. Patients classified as discharged were those who had achieved a level of self-management sufficient enough to discontinue clinic-based care [4], while undischarged patients were those who either continued treatment, returned for further intervention, or remained under clinical care beyond one year.

### 2.3 Data preprocessing

Prior to analysis, all collected data were subjected to exploratory data analysis (EDA) to evaluate variable distributions and detect patterns within the dataset. Bivariate analyses were conducted to identify correlations among variables, highlight key features, and assess potential regression relationships. Descriptive statistics were calculated to determine the extent to which variables conformed to a normal distribution. Outliers and missing values were identified during this process and were subsequently excluded from further analysis. Data attributes were transformed into appropriate analytical formats. Specifically, numeric features were standardized using a standard scaler (min–max scaling), while categorical and mixed-type variables were processed using one-hot encoding. For inferential statistical analysis, the Chi-square test was employed to compare categorical variables, and the Mann-Whitney U test was used for continuous variables. A two-sided *p*-value < 0.05 was considered to indicate statistical significance.

### 2.4 Machine learning algorithms, model development, and evaluation

A final dataset comprising 1050 patients was included in the analysis. Prior to model development, variables exhibiting high intercorrelation (Pearson’s correlation coefficient >0.7) were removed to minimize multicollinearity and enhance predictive accuracy.

A nested cross-validation (Nested CV) framework was implemented to ensure robust model evaluation and generalizability. This framework involved 10 independent random trials. The outer loop employed a 4-fold K-Fold cross-validation strategy, partitioning the dataset into 75% training and 25% testing subsets. Within each outer fold, an inner cross-validation loop was used to perform hyperparameter optimization via GridSearchCV (scikit-learn library [26]), with the objective of maximizing the receiver operating characteristic-area under the curve (ROC-AUC) on the training set.

Data imbalance was addressed using built-in balancing mechanisms specific to each algorithm, as well as through hyperparameter tuning strategies that incorporated the synthetic minority oversampling technique (SMOTE) and class-weighting adjustments. Seven machine learning algorithms were developed and evaluated: Decision Tree, Random Forest, Logistic Regression, K-Nearest Neighbors (KNN), Support Vector Classifier (SVC), Gradient Boosting, and Extreme Gradient Boosting (XGBoost). Model performance was assessed using multiple metrics, including accuracy, precision, recall, F1 score (micro and macro), and ROC-AUC.

For comparative model analysis, receiver operating characteristic (ROC) curves were generated for each algorithm. Mean true positive rates (TPRs) and false positive rates (FPRs) were calculated and averaged across all trials. The resulting aggregated mean ROC curves and their standard deviations were plotted to allow visual comparison of each model’s predictive performance. Furthermore, normalized confusion matrices were computed and visualized to evaluate the classification accuracy in greater detail.

To assess the relative importance of predictive features, two interpretability methods were employed: information gain and SHAP plots. For information gain, feature importance scores were computed by averaging the mean results obtained from 10 repeated trials. SHAP analysis was performed on the optimal model using the optimal hyperparameter configurations identified during model tuning, thereby offering interpretable insights into the contribution of each feature to the model’s predictions. Three types of SHAP plots were used to present the results: (1) bar plots showing the mean absolute SHAP value for each feature across all predictions, indicating the average magnitude of impact and enabling feature ranking; (2) SHAP summary plots (beeswarm plots), which depicted both the magnitude and direction of each feature’s effect; and (3) heatmaps that provided a visual overview of SHAP values for individual predictions across multiple features [12, 27].

### 2.5 Software and tools

Machine learning algorithms were implemented using the Python programming language (version 3.10.12, Python Software Foundation, Wilmington, DE, USA) within the Google Collaboratory notebook environment (Version 4, California, USA) [26]. All electronic devices utilized during the research process, including computers and storage drives, were secured with password protection. Data encryption protocols were employed to ensure the protection of sensitive information in accordance with standard data security practices, thereby maintaining participant confidentiality throughout the study.

## 3. Results

### 3.1 Patient demographics and clinical characteristics

From a retrospective review of 1237 patients newly diagnosed with TMD, 65 patients were excluded due to incomplete data, 49 patients were excluded based on the presence of other comorbid pain conditions, and 73 patients classified as improperly discharged were also excluded. This resulted in a final dataset of 1050 patients eligible for EDA, model development, and evaluation, as illustrated in Fig. 1.

**Fig. 1.** Flowchart of all TMD patients for model analysis. EDA: Exploratory Data Analysis; TMD: Temporomandibular Disorders.

The mean age of the included patients was 33.02 ± 16.67 years. The cohort exhibited a female predominance, with 779 females (74.19%) and 271 males (25.81%). Additional clinical and demographic characteristics, stratified by discharge status, are summarized in Table 2. Quantitative variables are presented as mean ± standard deviation (SD), while categorical variables are reported as frequencies and percentages. Given the non-normal distribution of the data, the Mann-Whitney U test was applied for comparisons involving continuous variables, and the Chi-square test was used for categorical variables, as shown in Table 2.

**Table 2.** Demographic, clinical, and diagnostic characteristics of TMD patients stratified by treatment outcome (discharged *vs*. undischarged).

Variable	Subgroups	Total		Undischarged group		Discharged group		*p*-value
		(n = 1050)	(%)	(n = 169)	(%)	(n = 881)	(%)	
Demographic data								
Gender								
	Male	271	25.81	39	23.08	232	26.33	0.4293
	Female	779	74.19	130	76.92	649	73.67	
Age in years (minimum–maximum)	-	9–87		11–85		9–87		-
Age in years (mean ± SD)	-	33.02 ± 16.67		32.16 ± 15.73		33.19 ± 16.85		0.5068
Occupation								
	Student/no job	583	55.52	93	55.03	490	55.62	0.8250
	Managers	48	4.57	8	4.73	40	4.54	
	Professionals	141	13.43	20	11.83	121	13.74	
	Technicians	35	3.33	5	2.96	30	3.40	
	Clerical workers	63	6.00	14	8.28	49	5.56	
	Service workers	89	8.48	13	7.69	76	8.63	
	Agricultural workers	6	0.57	0	0.00	6	0.68	
	Craft workers	17	1.62	2	1.19	15	1.70	
	Machine operators	3	0.29	0	0.00	3	0.34	
	Elementary workers	54	5.14	12	7.10	42	4.77	
	Armed forces	11	1.05	2	1.19	9	1.02	
Clinical data								
Numeric rating scale (mean ± SD)	-	6.45 ± 2.81		7.27 ± 2.50		6.30 ± 2.84		<0.0001
Anxiety (mean ± SD)	-	6.46 ± 3.64		7.86 ± 4.60		6.19 ± 3.36		<0.0001
Depression (mean ± SD)	-	4.08 ± 3.31		5.06 ± 4.14		3.89 ± 3.09		0.0020
Stress (mean ± SD)	-	14.33 ± 9.37		18.74 ± 12.49		13.48 ± 8.39		<0.0001
Body mass index (mean ± SD)	-	22.14 ± 4.12		21.93 ± 3.83		22.18 ± 4.18		0.4619
Sleep problem								
	No	609	58.00	77	45.56	532	60.39	0.0004
	Yes	441	42.00	92	54.44	349	39.61	
Psychiatric problem								
	No	967	92.10	133	78.70	834	94.67	<0.0001
	Yes	83	7.90	36	21.30	47	5.33	
Clenching								
	No	687	65.43	102	60.36	585	66.41	0.1540
	Yes	363	34.57	67	39.64	296	33.59	
Bruxism								
	No	643	61.24	94	55.62	549	62.32	0.1211
	Yes	407	48.76	75	45.38	332	37.68	
Onset duration (mean ± SD)	-	16.59 ± 35.36		21.13 ± 36.42		15.73 ± 35.10		0.0003
Neck pain								
	No	423	41.14	51	30.18	372	42.44	0.0045
	Yes	627	58.86	118	69.92	509	57.78	
Back pain								
	No	531	50.57	79	46.75	452	51.31	0.3163
	Yes	519	49.43	90	53.25	429	48.69	
Poor posture								
	No	381	36.29	61	36.09	320	36.32	1.0000
	Yes	669	63.71	108	63.91	561	63.68	
Irritable bowel syndrome								
	No	936	89.14	143	84.62	793	90.01	0.0536
	Yes	114	10.86	26	15.38	88	9.99	
Itchy skin								
	No	995	94.76	162	95.86	833	94.55	0.6102
	Yes	55	5.24	7	4.14	48	3.45	
Tinnitus								
	No	940	89.52	147	86.98	793	90.01	0.2980
	Yes	110	10.48	22	13.02	88	9.99	
Tension-type headache								
	No	736	70.10	108	63.91	628	71.28	0.0677
	Yes	314	20.90	61	36.09	253	28.72	
Migraine								
	No	892	84.95	139	82.25	753	85.47	0.3392
	Yes	158	15.05	30	17.75	128	14.53	
Trauma event								
	No	876	83.43	135	79.88	741	84.11	0.2146
	Yes	174	16.57	34	20.12	140	15.09	
Hypertension								
	No	992	94.48	166	98.22	826	93.76	0.0319
	Yes	58	5.52	3	1.78	55	6.24	
Thyroid disease								
	No	1019	97.05	163	96.45	856	97.16	0.8000
	Yes	31	2.95	6	3.55	25	2.84	
Exercise								
	No	781	74.38	131	77.51	650	73.78	0.3562
	Yes	269	23.62	38	22.49	231	26.22	
Splint use								
	No	1024	97.52	162	95.86	862	97.84	0.2109
	Yes	26	2.48	7	4.14	19	2.16	
Diagnosis								
	Pain-related conditions	469	44.67	76	44.97	393	44.61	0.3929
	Structural abnormalities	68	6.47	7	4.14	61	6.92	
	Combined conditions	513	48.76	86	50.89	427	48.47	

*SD: standard deviation.*

### 3.2 Correlation and multicollinearity analysis of variables

EDA revealed correlations among both continuous and binary variables in the dataset. As illustrated in Fig. 2A, two prominent correlations were observed among the continuous variables: anxiety and stress levels (correlation coefficient = 0.78), and anxiety and depression levels (correlation coefficient = 0.68). Among the binary variables, shown in Fig. 2B, the strongest association was identified between back pain and neck pain (correlation coefficient = 0.51). Due to the high correlation between stress and anxiety levels (correlation coefficient >0.7), the stress variable was excluded from further analysis to minimize redundancy. This resulted in a refined dataset consisting of 36 variables for model development. Despite these observed correlations, multicollinearity was not deemed problematic. Variance inflation factor (VIF) analysis indicated that all variables remained within acceptable limits, with the highest VIF value being 3.59 for the neck pain variable. Since VIF values exceeding 10 are typically indicative of problematic multicollinearity, the results confirm that multicollinearity was within acceptable thresholds in the dataset.

**Fig. 2.** Correlation matrix of variables. The gradient color scale represents the strength of the correlation coefficients for (A) continuous variables and (B) binary variables. Stronger correlations are indicated by darker shades. BMI: Body Mass Index; NRS: Numeric Rating Scale.

### 3.3 Evaluation of model performance

The performance metrics of all machine learning algorithms are summarized in Table 3. Model evaluation primarily relied on ROC-AUC, due to its robustness in assessing classifier performance in the presence of class imbalance. Among the evaluated models, the Random Forest algorithm demonstrated the highest overall predictive performance based on ROC-AUC. It achieved an accuracy of 0.7901 ± 0.0383. The precision for predicting undischarged and discharged patients was 0.3512 ± 0.1239 and 0.8611 ± 0.0193, respectively. Recall values were 0.2459 ± 0.0891 for the undischarged group and 0.8943 ± 0.0549 for the discharged group. The model yielded an F1 score (micro) of 0.7901 ± 0.0383 and an F1 score (macro) of 0.5709 ± 0.0254. Class-specific F1 scores were 0.2654 ± 0.0584 for the undischarged group and 0.8764 ± 0.0263 for the discharged group. The corresponding ROC-AUC was 0.6442 ± 0.0455. In contrast, the KNN model exhibited the lowest ROC-AUC despite a relatively higher accuracy. It achieved an accuracy of 0.8336 ± 0.0170, with precision values of 0.4321 ± 0.2042 for the undischarged group and 0.8456 ± 0.0192 for the discharged group. However, recall for the undischarged group was notably low at 0.0655 ± 0.0338, compared to 0.9810 ± 0.0123 for the discharged group. The F1 score (micro) was 0.8336 ± 0.0170, and the F1 score (macro) was substantially lower at 0.5092 ± 0.0268. Class-specific F1 scores were 0.1103 ± 0.0519 for the undischarged group and 0.9081 ± 0.0102 for the discharged group. The model’s ROC-AUC was 0.5717 ± 0.0490.

**Table 3.** Comparison of model performance matrices.

		Decision Tree	Random Forest	Logistic Regression	KNN	SVC	Gradient Boosting	XGBoost
Accuracy		0.5999 ± 0.0724	0.7901 ± 0.0383	0.6312 ± 0.0313	0.8336 ± 0.0170	0.6425 ± 0.0528	0.8381 ± 0.0189	0.6861 ± 0.0448
Precision								
	Undischarged group	0.2843 ± 0.0428	0.3512 ± 0.1239	0.2273 ± 0.0375	0.4321 ± 0.2042	0.2319 ± 0.0407	0.4564 ± 0.2915	0.2503 ± 0.0552
	Discharged group	0.8662 ± 0.0279	0.8611 ± 0.0193	0.8805 ± 0.0228	0.8456 ± 0.0192	0.8779 ± 0.0195	0.8471 ± 0.0216	0.8757 ± 0.0236
Recall								
	Undischarged group	0.4999 ± 0.1199	0.2459 ± 0.0891	0.5388 ± 0.0838	0.0655 ± 0.0338	0.5088 ± 0.0884	0.0737 ± 0.0532	0.4564 ± 0.1025
	Discharged group	0.6191 ± 0.1037	0.8943 ± 0.0549	0.6488 ± 0.0408	0.9810 ± 0.0123	0.6660 ± 0.0728	0.9853 ± 0.0166	0.7317 ± 0.0666
	F1 (micro)	0.5999 ± 0.0724	0.7901 ± 0.0383	0.6312 ± 0.0313	0.8336 ± 0.0170	0.6425 ± 0.0528	0.8381 ± 0.0189	0.6861 ± 0.0448
	F1 (macro)	0.5005 ± 0.0417	0.5709 ± 0.0254	0.5322 ± 0.0228	0.5092 ± 0.0268	0.5349 ± 0.0299	0.5154 ± 0.0413	0.5554 ± 0.0362
F1								
	Undischarged group	0.2843 ± 0.0428	0.2654 ± 0.0584	0.3181 ± 0.0473	0.1103 ± 0.0519	0.3148 ± 0.0432	0.1201 ± 0.0793	0.3159 ± 0.0534
	Discharged group	0.7167 ± 0.0741	0.8764 ± 0.0263	0.7462 ± 0.0282	0.9081 ± 0.0102	0.7575 ± 0.0509	0.9107 ± 0.0113	0.7950 ± 0.0357
	ROC-AUC	0.5840 ± 0.0546	0.6442 ± 0.0455	0.6370 ± 0.0416	0.5717 ± 0.0490	0.6285 ± 0.0349	0.6283 ± 0.0415	0.6369 ± 0.0458

*KNN: K-Nearest Neighbors; ROC-AUC: Receiver Operating Characteristic-Area Under the Curve; SVC: Support Vector Classifier; XGBoost: Extreme Gradient Boosting.*

The normalized confusion matrices presented in Fig. 3 illustrate the average classification performance of each machine learning model across 10 repeated experiments, highlighting their ability to differentiate between class 0 (undischarged group) and class 1 (discharged group). The Gradient Boosting and KNN classifiers showed excellent performance in identifying discharged patients, achieving true positive rates of 99% and 98%, respectively. However, both models demonstrated poor sensitivity in detecting the undischarged group, with correct classification rates of only 7%. The Random Forest model also performed well for the discharged group, correctly identifying 89% of those cases, while achieving a higher recall for the undischarged group at 25%. Notably, the XGBoost model demonstrated more balanced classification, with 73% of discharged patients and 45% of undischarged patients correctly identified.

**Fig. 3.** Normalized confusion matrices of all machine learning models for classifying discharged (class 1) and undischarged (class 0) TMD patients. The values represent the average of 10 repeated experiments for each model. Each matrix displays the proportion of correctly and incorrectly classified instances within each class, providing a visual summary of classification performance. KNN: K-Nearest Neighbors; SVC: Support Vector Classifier; XGBoost: Extreme Gradient Boosting.

In contrast to models with high-class imbalance, SVC exhibited more balanced classification performance across both outcome groups. Logistic Regression correctly identified 54% of patients in the undischarged group and 65% in the discharged group. Similarly, SVC achieved classification rates of 52% and 67% for the undischarged and discharged groups, respectively. The Decision Tree model demonstrated the weakest performance overall, correctly classifying only 50% of undischarged and 62% of discharged patients.

Fig. 4 presents the average ROC-AUC values for all machine learning models. Based on results from the nested CV framework, Logistic Regression yielded a mean ROC-AUC of 0.64 ± 0.04, while Random Forest achieved a comparable mean ROC-AUC of 0.64 ± 0.05, albeit with a slightly higher standard deviation. Among the models evaluated, these two demonstrated the most consistent and reliable predictive performance across the outer validation fold.

**Fig. 4.** Comparison of average receiver operating characteristic (ROC) curves for all machine learning models, derived from nested cross-validation (Nested CV). Each curve represents the mean performance across 10 repeated experiments. The area under the curve (AUC) and corresponding standard deviation are indicated in the legend. The diagonal line denotes the performance of a random classifier (AUC = 0.5). KNN: K-Nearest Neighbors; SVC: Support Vector Classifier; XGBoost: Extreme Gradient Boosting.

### 3.4 Feature important analysis

#### 3.4.1 Information gain-based feature ranking

The ranking of predictive features based on information gain analysis is presented in Fig. 5A. In the Random Forest model, the five most informative predictors were anxiety level, onset duration, BMI, pain severity, and age, with feature importance scores of 0.1187, 0.1175, 0.1107, 0.1052, and 0.0939, respectively. These features contributed most significantly to the model’s ability to distinguish between discharged and undischarged patients.

#### 3.4.2 SHAP value of feature importance

The SHAP-based feature importance results for the Random Forest model are shown in Fig. 5B. In this bar plot, features with higher absolute SHAP values, reflecting greater average impact on model predictions, are ranked at the top [27]. The most influential feature was onset duration, which had the highest mean SHAP value of 0.05. This was followed by pain severity (mean SHAP = 0.03), anxiety level (mean SHAP = 0.03), presence of neck pain (mean SHAP = 0.02), and BMI (mean SHAP = 0.02). Positive SHAP values for these variables indicated that they contributed to classifying a patient into the discharged group. Notably, while these top-ranked features showed substantial individual importance, the aggregate contribution of the remaining lower-ranked features collectively accounted for the highest cumulative impact, with a combined mean SHAP value of 0.06. Furthermore, there was complete overlap between the top nine features identified by SHAP analysis and those ranked highly in the information gain method, which included anxiety level, onset duration, BMI, pain severity, age, depression level, presence of psychiatric problems, sleep problems, and neck pain.

**Fig. 5.** Visualization of feature importance in the Random Forest model. (A) Bar chart displaying the ranking of predictive features based on information gain scores. (B) Bar chart showing the mean absolute SHAP values for each predictor, indicating their relative contribution to classification into the discharged group. BMI: Body Mass Index; NRS: Numeric Rating Scale; SHAP: Shapley Additive exPlanations.

SHAP beeswarm plot shown in Fig. 6A illustrates the distribution and individual contributions of features to the predictive outcomes generated by the Random Forest model. Each dot represents a Shapley value for a single feature in one patient case. Feature names are presented on the y-axis, while the x-axis denotes the magnitude and direction of each Shapley value. The color gradient of each point reflects the actual feature value, ranging from low (blue) to high (pink), and the vertical dispersion highlights the variability of feature effects across the dataset. Features are ranked in descending order of overall importance [27]. Notably, shorter onset duration, lower pain severity, and reduced anxiety levels were predominantly associated with positive SHAP values, indicating a higher likelihood of discharge. In addition, the absence of neck pain and higher BMI, ranked fourth and fifth, respectively, also contributed positively to discharge predictions. The SHAP summary heatmap in Fig. 6B provides further insight into the influence of key predictors across individual patients. In this visualization, each row corresponds to a feature, while each column represents a single patient instance. The color intensity reflects the magnitude and direction of the SHAP value, with pink indicating a positive contribution to the prediction (*i.e.*, discharged group) and blue indicating a negative contribution (*i.e.*, undischarged group). This heatmap highlights the consistent importance of onset duration, pain severity, and anxiety level, which exhibit strong contributions across multiple cases [27]. In contrast, features such as neck pain and BMI showed consistent but more moderate influence on model predictions.

**Fig. 6.** SHAP value distribution and feature contributions in the Random Forest model for predicting discharge outcomes. (A) Beeswarm plot showing the direction and magnitude of each feature’s impact on the model output for the discharged group. Each dot represents a single patient; the horizontal axis indicates the SHAP value, while the color denotes the actual feature value (pink for high and blue for low). Features are listed in order of their overall importance. (B) SHAP summary heatmap displaying feature contributions to model predictions across individual patient cases. Each row corresponds to a feature, and each column to a patient. Pink indicates a positive SHAP value (favoring discharge prediction), while blue indicates a negative SHAP value (favoring undischarged prediction). BMI: Body Mass Index; NRS: Numeric Rating Scale; SHAP: Shapley Additive exPlanations.

## 4. Discussion

### 4.1 Prognostic factors influencing TMD patient readiness for discharge

To our knowledge, this is the first study to investigate prognostic factors influencing treatment response based on readiness for discharge in patients with TMD managed at a tertiary orofacial pain clinic, utilizing advanced machine learning methodologies. By using SHAP value-based interpretation within the machine learning framework, we identified key predictors that contribute to a patient’s likelihood of achieving discharge readiness [28]. Previous studies have highlighted the role of psychological factors [9], pain intensity [29], and biopsychosocial factors [5] as significant predictors of treatment response in TMD patients. In our analysis, onset duration emerged as the most influential predictor across multiple machine learning models in determining patient readiness for discharge. Pain severity was identified as the second most important variable, likely reflecting its direct impact on functional status and overall recovery trajectory. Anxiety level, as an indicator of psychological well-being, also played a key role in capturing both the emotional and physical dimensions of TMD. Additionally, the presence of neck pain, which is frequently associated with orofacial and masticatory dysfunction, emphasized the interconnected nature of symptom presentation in TMD patients [23]. BMI also emerged as a relevant predictor, suggesting a potential association between lifestyle or systemic factors and the chronicity of TMD symptoms. In addition, the cumulative influence of numerous additional features, quantified through SHAP values, further reinforces the multifactorial and heterogeneous nature of TMD, which collectively shapes patients’ readiness for discharge.

#### 4.1.1 Onset duration

Our findings highlighted the significant influence of symptom onset timing on discharge outcomes in patients with TMD. Individuals who sought treatment shortly after the onset of symptoms were more likely to develop self-management capabilities and achieve favorable outcomes [30]. In contrast, a prolonged interval between symptom onset and clinical consultation was associated with extended treatment duration before discharge [7]. Such delays may contribute to central sensitization, increased psychological distress, and heightened frustration resulting from persistent symptoms.

Patients experiencing chronic symptoms often consult multiple healthcare providers, a pattern reflecting dissatisfaction and diminished confidence in recovery prospects [7, 30, 31]. In the present study, most patients reported an onset duration of less than one year, indicating that professional care was generally sought after unsuccessful self-management or when the emotional burden of symptoms became unmanageable [30, 31]. For those presenting earlier, the prognosis appeared more favorable, supporting the importance of timely intervention in mitigating chronicity and improving discharge readiness [30].

#### 4.1.2 Pain severity

Pain severity was identified as an essential determinant of both short- and long-term patient outcomes. In this study, shorter onset duration was associated with a shorter overall treatment duration. A prospective cohort study similarly reported that individuals whose pain did not substantially interfere with daily functioning during the initial phase were more likely to engage in and sustain self-care behaviors over time [3]. The way in which patients perceived and experienced their pain influenced not only their immediate coping strategies but also their broader psychological outlook and quality of life [31]. Cultivating a sense of reassurance, specifically, the belief that pain would remain manageable, was found to support adherence to self-care strategies and facilitate discharge readiness [4]. Notably, several studies have demonstrated that higher pain intensity at onset may increase the likelihood of chronic pain development by up to 50% [4, 7, 29]. Findings from the Orofacial Pain Prospective Evaluation and Risk Assessment (OPPERA) study further support this association, showing that greater pain severity, increased pain frequency, and longer symptom duration were predictive of persistent TMD. In addition, patients with higher baseline pain tended to exhibit poorer psychosocial profiles based on DC/TMD Axis II assessments. At follow-up, this subgroup demonstrated worsened psychological functioning and reduced jaw mobility [7]. These findings highlight the importance of early pain identification and intervention, as timely and adequate pain management may help prevent chronic progression and support improved long-term outcomes.

#### 4.1.3 Anxiety level

Anxiety level consistently ranked among the most influential predictors in both SHAP and information gain analyses, indicating that psychosocial variables substantially affect treatment outcomes in TMD patients. Previous studies have identified baseline anxiety as a premorbid risk factor, with higher anxiety levels associated with heightened symptom perception and poorer treatment responses [23, 30]. From a physiological perspective, anxiety may contribute to dysregulation of the hypothalamic-pituitary-adrenal (HPA) axis, leading to increased sympathetic activation, elevated muscle tension, and central sensitization [9, 30]. A cross-sectional study conducted in the United Kingdom further reported that chronic anxiety can undermine patient confidence, thereby reducing engagement in self-care and exacerbating symptom chronicity [4]. These findings suggest that addressing anxiety early in the course of treatment may improve outcomes and facilitate readiness for discharge [3].

#### 4.1.4 Neck pain

The presence of neck pain was also identified as a relevant prognostic feature, likely due to the anatomical and neurophysiological interactions between the cervical and orofacial regions. Convergence between the trigeminal and cervical nerve systems has been reported to intensify pain perception in TMD patients [23, 32]. This overlap was further supported by mechanisms such as central sensitization, impaired descending pain inhibition, and convergent neuronal signaling [23, 32, 33]. A randomized controlled trial demonstrated that neck control training in patients with TMD could improve pain, jaw function, and oral health-related quality of life, potentially by stimulating cortical neuroplasticity and reorganizing altered motor function [32]. Additionally, several studies have shown an association between forward head posture and TMD, whereby shortening of the sternocleidomastoid and posterior cervical extensor muscles could alter mandibular positioning and head orientation. These postural changes may propagate along the myofascial chains, influencing overall body posture [34, 35].

#### 4.1.5 BMI

BMI is a commonly used measure for assessing body composition, with lower values reflecting reduced body mass relative to height [35]; however, the association between BMI and TMD risk remains inconclusive [19]. In this study, the mean BMI of the undischarged group was lower than that of the discharged group, contrasting with earlier findings that linked obesity with increased risk of persistent pain conditions [29]. However, a systematic review suggested that higher BMI may have a protective effect against TMD, whereby individuals with higher BMI demonstrated better masticatory performance, including greater bite force and longer chewing cycle duration, functions often impaired in TMD patients, who typically exhibit an anterior shift in occlusal force distribution [19]. Similarly, a randomized study further supported a causal relationship between genetically predicted low BMI and increased susceptibility to TMD [35]. In population-based studies from South Korea, women diagnosed with TMD had significantly lower BMI compared to controls, suggesting a possible sex-specific vulnerability [36]. Moreover, a Japanese study found that individuals with lower BMI were more prone to persistent pain. This association may reflect physiological factors such as reduced muscle mass or physical inactivity. It is also plausible that lower BMI in undischarged patients may result from diminished appetite or limited food intake due to masticatory pain or stress-related responses [37].

### 4.2 Toward a holistic model of TMD management

In this tertiary care setting, discharge decisions were jointly determined by the attending clinician and the patient, and documented in the medical records. Criteria influencing discharge often included tolerable pain levels, meaningful improvement in jaw function, or the patient’s ability to adapt to residual symptoms such as TMJ clicking. This study aimed to capture the patient’s subjective response to treatment, while simultaneously evaluating objective clinical parameters as prognostic indicators. Although objective metrics have traditionally dominated assessments of treatment success or failure, emerging evidence suggests that subjective satisfaction may persist despite minimal change in quantifiable outcomes [38, 39].

The findings of this study underscore the multidimensional nature of TMD, wherein multiple factors, rather than any single variable, contribute to a patient’s readiness for discharge. From a neurobiological perspective, pain is shaped by both physiological and psychological processes, reinforcing the need for a comprehensive, biopsychosocial approach to clinical management [1, 23]. Historically, chronic pain treatment models have prioritized the resolution of primary symptoms, often at the expense of adequately addressing psychosocial dimensions during routine consultations [31]. However, there is an increasing number of studies that support the use of integrative care models that concurrently address emotional, social, and physical health domains within a single clinical encounter [3, 6]. A stronger doctor-patient relationship, coupled with a focus on functional well-being and psychological coping capacity rather than symptom severity alone, may foster more effective and collaborative chronic pain management. This patient-centered approach promotes self-awareness, resilience, and active engagement in self-management practices [30]. Adopting such a holistic framework in TMD care may thus contribute to more sustainable outcomes and improved quality of life.

Our findings indicated that 16.10% of patients experienced persistent TMD, preventing them from being discharged, suggesting that most (*i.e.*, nearly 84%) were able to manage or recover from their symptoms, aligning with the fluctuating nature of TMD, in which pain-free intervals often exceed periods of active symptoms [2]. As a result, any subsequent reduction in symptoms may partly reflect natural fluctuations rather than treatment effects, a phenomenon known as regression to the mean. This interpretation is further supported by epidemiological studies, which report that 15–20% of TMD cases exhibit severe symptoms as measured by the Graded Chronic Pain Scale, while only approximately 10% of adolescents experience worsening pain over time [3]. These findings suggest that TMD symptoms often resolve when patients are able to clearly identify symptom triggers and engage in effective self-care strategies [3, 9]. A key element in this process is self-confidence, as evidenced by studies demonstrating that clear explanations of TMD pathophysiology, combined with the identification and reduction of aggravating factors, can alleviate symptom burden [6]. Therefore, by providing patients with knowledge and reinforcing self-belief, clinicians may facilitate more effective self-management and increase the likelihood of long-term recovery.

### 4.3 Enhancing model performance

Some models in this study, including Random Forest, KNN, and Gradient Boosting, demonstrated high accuracy but only moderate ROC-AUC values. This discrepancy is commonly observed in the context of imbalanced datasets, where prediction bias favors the majority class. A major challenge encountered in the machine learning pipeline was overfitting, which reduced the generalizability and predictive accuracy of certain models. Specifically, Gradient Boosting and KNN showed a pronounced tendency to favor predictions toward the discharged group, likely due to class imbalance or overfitting to the dominant class. While these models performed well in identifying discharged patients, they frequently misclassified undischarged cases—a pattern similarly noted with the Random Forest model.

In contrast, the XGBoost classifier yielded a more balanced performance between groups, although its overall predictive capacity remained suboptimal. Logistic Regression and SVC provided the most balanced classification results, demonstrating less bias and potentially greater suitability in scenarios where equal attention is required for both outcome groups. Meanwhile, the Decision Tree model exhibited limited discriminative power, possibly due to overfitting or inadequate model complexity. To improve model performance, several strategies were employed. Hyperparameter tuning was applied to optimize model-specific settings; for example, varying the depth of tree-based models allowed identification of the appropriate level of complexity to avoid overfitting [26]. Another essential technique was feature selection, which was used to eliminate irrelevant or redundant variables, thereby reducing noise and enhancing overall accuracy [9]. Finally, managing data imbalance was essential to ensuring reliable predictions. Techniques such as oversampling, undersampling, or the use of algorithms designed to address imbalance, such as SMOTE or class-weight adjustments, were effective in reducing the risks of biased predictions. In this study, class-weight adjustment was implemented to account for data imbalance, contributing to more reliable and equitable model performance.

#### 4.3.1 Feature reduction

Prior research has demonstrated that the removal of selected variables can enhance predictive accuracy in machine learning models [9]. For instance, the variable stress was excluded in our present study due to its strong correlation with anxiety, which has been consistently identified as a significant factor in the onset and progression of TMD [1, 23, 30]. However, the decision to remove stress variable also illustrated a key trade-off in predictive modeling, balancing the inclusion of potentially informative predictors with the need for model simplicity and performance optimization. In both clinical and research settings, such decisions should be informed not only by statistical considerations but also by the clinical relevance and interpretability of each variable. Achieving this balance is essential for developing models that are not only statistically robust but also practically applicable in guiding treatment decisions and informing prognostic evaluations.

#### 4.3.2 Managing class imbalance

Persistent TMD represents a relatively small proportion of cases in this cohort, resulting in an imbalanced dataset that, if unaddressed, may bias model predictions. To mitigate such effects, several strategies were implemented. One approach involved the use of balanced algorithms, which assign differential weights to positive and negative classes to rebalance their influence on model outputs. Additionally, in-algorithm techniques such as the SMOTE were incorporated into tree-based models to increase the representation of the minority class [40]. Furthermore, the use of four-fold cross-validation further ensured that each subset contained an adequate number of persistent TMD cases, thereby enhancing both model training and evaluation. Collectively, these methods contributed to more reliable and less biased predictive performance.

ROC-AUC was used as the primary metric for evaluating model performance in the presence of class imbalance. It quantifies the model’s ability to distinguish between classes by plotting the true positive rate (sensitivity) against the false positive rate (1 − specificity) across all possible classification thresholds. Unlike accuracy, which can be misleading when classes are imbalanced, ROC-AUC does not depend on a single cutoff and provides a more comprehensive assessment of model discrimination. This characteristic is particularly useful in datasets with a smaller proportion of persistent TMD cases, where minimizing false negatives or false positives may be prioritized differently based on clinical objectives [41, 42]. Moreover, because ROC-AUC accounts for the model’s performance across both classes, it helps ensure that predictions are not dominated by the majority class, reducing the risk of underrepresenting the minority group [41]. Thus, ROC-AUC supports the selection of models with superior discriminatory ability and enables informed threshold adjustments to achieve an appropriate balance between sensitivity and specificity.

### 4.4 Feature importance illustrations using information gain or SHAP

Information gain analysis ranks features based on their capacity to reduce uncertainty in predicting the target variable. A higher information gain indicates a greater contribution of that feature to the prediction, and the metric is calculated using entropy, which measures the level of uncertainty within the dataset. However, a known limitation of this approach is its tendency to favor features with many unique values, which may appear predictive even if their actual contribution is minimal. In addition, in datasets containing noise, entropy estimates may become unstable, potentially leading to suboptimal decision splits [43].

To overcome these limitations and obtain a more nuanced understanding of feature contributions, SHAP analysis was also applied. SHAP is grounded in the cooperative game theory and provides contrastive explanations by comparing individual predictions to the average model output, distributing attribution fairly among input features [44]. One of SHAP’s strengths lies in the interpretability of its values, which are expressed in the same units as the model output. SHAP facilitates both global interpretation, through feature importance rankings, dependence plots, interaction effects, clustering, and summary plots, and local interpretability at the level of individual predictions. Despite these advantages, SHAP also has limitations. Calculating exact Shapley values across many instances can be computationally demanding, and global SHAP analyses require aggregation over a large number of samples to ensure stability. Moreover, in the presence of multicollinearity, SHAP may assign disproportionately high importance to statistically improbable combinations of features, potentially complicating interpretation [27].

### 4.5 Strengths and limitations

A major strength of this study was the setting in a tertiary care center, which enabled access to a substantial number of patients with chronic TMD. This environment facilitated specialist-led clinical assessments, thereby ensuring a higher level of diagnostic accuracy compared to studies relying solely on self-reported symptoms. The application of machine learning further enhanced the study’s analytical depth, allowing for the identification of complex relationships between variables that may extend beyond conventional expert assumptions while reducing subjective bias. Additionally, machine learning techniques are often superior to traditional statistical methods in capturing non-linear associations, thus providing a more nuanced understanding of the dataset.

However, this study also has several limitations. First, the findings may not be generalizable to broader TMD populations, particularly those of non-Asian ethnicities. Furthermore, patients in tertiary care settings often present with more complex conditions and higher expectations for treatment, which may not reflect the clinical profiles typically seen in primary care. The inherent class imbalance in TMD outcomes, where most patients are ultimately discharged, may also bias the predictive models toward the majority class, thereby resulting in moderate overall model performance. Nonetheless, the predictors identified in this study may still serve as a useful screening tool for general practitioners to identify potentially complicated cases. These findings are consistent with those of the prospective OPPERA study, which similarly investigated prognostic indicators in persistent TMD patients [7]. The retrospective design posed additional challenges, including susceptibility to recall bias, variability in documentation quality, and missing data, all of which may compromise data integrity. Moreover, because formal discharge criteria were not uniformly established, discharge status in this study was determined based on documentation in the medical records, incorporating both professional judgment and patient preference. Future prospective or multi-center studies utilizing standardized data collection protocols would help mitigate these limitations and improve the external validity of TMD research.

### 4.6 Future recommendations

Future studies should aim to improve both the generalizability and predictive performance of the models by expanding the dataset to include patients from primary care settings, thereby capturing a more heterogeneous patient population and broader treatment contexts. Additionally, increasing the representation of non-discharged patients is essential to address class imbalance and enhance model discrimination. Clinical variables with stronger associations to discharge readiness, such as structure-specific pain (*e.g.*, myalgia or arthralgia) and the 20-item checklist of general symptoms, should be incorporated, as supported by findings from the prospective OPPERA study [7]. Model performance may also benefit from a two-stage approach that integrates expert clinical judgment with the best-performing, well-balanced algorithm, such as the Logistic Regression model.

## 5. Conclusions

This study applied machine learning methods, with SHAP analysis for interpretability, to identify key factors associated with treatment response in patients with TMD. Onset duration, pain severity, anxiety level, presence of neck pain, and BMI emerged as the most influential predictors of discharge readiness, reflecting favorable clinical outcomes. Among the evaluated models, Random Forest achieved the highest predictive performance, with a maximum ROC-AUC of 0.6442. These findings may support clinical decision-making by identifying patients at increased risk of persistent symptoms and informing individualized treatment strategies.
